# The effect of fibrin glue on the early healing phase of intestinal anastomoses in the rat

**DOI:** 10.1007/s00384-012-1435-5

**Published:** 2012-03-08

**Authors:** Rozemarijn J. van der Vijver, Cees J. H. M. van Laarhoven, Ben M. de Man, Roger M. L. M. Lomme, Thijs Hendriks

**Affiliations:** Department of Surgery, Radboud University Nijmegen Medical Centre, PO Box 9101, 6500 HB Nijmegen, The Netherlands

**Keywords:** Anastomoses, Intestine, Fibrin glue, Rat, Seal

## Abstract

**Purpose:**

Protecting the anastomotic integrity using suture or staple line reinforcement remains an important goal for ongoing research. The present comprehensive study aims to establish the effects of fibrin glue on the early phase of anastomotic healing in the rat intestine.

**Methods:**

One hundred and eight young adult male Wistar rats underwent resection and anastomosis of both the ileum and colon. In half, fibrin glue was applied around the anastomoses. Parameters for repair included wound strength, both bursting pressure and breaking strength at days 1, 3, and 5 after operation; hydroxyproline content; and histology, the latter also after 7 days.

**Results:**

A transient colonic ileus was observed in the experimental group. Anastomotic breaking strength was always similar in both the control and fibrin glue groups. Anastomotic bursting pressures remained low at days 1 and 3, without any differences between the groups. In both groups, the bursting pressure increased sharply (*p* < 0.001) between days 3 and 5. At day 5, the bursting pressure in the fibrin glue group remained below than that in the controls, although only significantly (*p* = 0.0138) so in the ileum. At day 5, but not at day 7, the wounds in the fibrin glue group contained less collagen. Other aspects of microscopic wound architecture appeared to be the same.

**Conclusions:**

There is no justification for using fibrin glue on patent anastomoses constructed under low-risk conditions. Its potential benefit under conditions where chances for anastomotic leakage are enhanced needs further investigation.

## Introduction

Anastomotic dehiscence is the most feared and potentially devastating complication after gastrointestinal surgery. A leaking anastomosis is associated with increased morbidity and considerable mortality [1, 2]. Protecting the anastomotic integrity and finding ways to minimize, postpone, or prevent the consequences of leakage, therefore, remain as the important goals of ongoing research. In theory, it seems logical to apply some kind of seal around the anastomosis to prevent leakage. Very recently, it has been emphasized again that the potential benefits of such suture or staple line reinforcement need investigation [3]. In this respect, a possible role for tissue glues should not be overlooked.

Tissue adhesives or glues, and in particular fibrin glue, are already used in the different fields of surgery to promote tissue union, sealing, hemostasis, and wound healing [4–7]. Fibrin glue consists of homologous plasma-derived fibrin products from pooled donors combined with bovine thrombin, and mimics the final stages of blood coagulation and fibrin clot formation. While the concept that fibrin glue may be used to strengthen the intestinal anastomosis [8, 9] is certainly not new, it has not led to consistent and reliable data which demonstrate its effectiveness and supply indications for its use. So far, clinical data are mainly limited to a few observational reports involving anastomoses in gastric bypass patients, which might indicate a potential benefit in terms of a reduced frequency of leakage [10–13]. Very recently, a similar suggestion has been reported for stapled anastomoses during laparoscopic resection of rectal cancer [14]. The question still stands if the routine application of fibrin glue to sutured or stapled intestinal anastomoses may contribute to successful healing. Incidental data from preclinical studies seem to suggest that fibrin glue applied to patent sutured colonic anastomoses can negatively affect their strength [15, 16]. If true, such an effect would limit the range of possible indications for its clinical use. In order to evaluate if fibrin glue can indeed have a relevant negative effect on anastomotic healing in the intestine, we conducted a comprehensive experimental study in the rat, examining anastomoses in both the ileum and colon and measuring multiple parameters for wound repair on three different days during the early crucial phase of healing.

## Methods

### Experimental design

One hundred and eight male Wistar rats, weighing 250–295 g (Harlan BV, Horst, The Netherlands), were housed two per cage and accustomed to laboratory conditions for 5 days before the start of the experiment. They were randomly divided over the control group (*n* = 54) or the group in which fibrin glue was applied around the anastomoses (*n* = 54). All rats underwent intestinal resection, and anastomoses were constructed in both the colon and ileum. Within each group, 16 animals were sacrificed on days 1, 3, and 5 after surgery and 6 animals 7 days after operation. All animals were observed closely and weighed daily and had free access to water and standard rodent chow throughout the entire experimental period (Hope Farms, Woerden, The Netherlands). The Animal Ethics Review Committee of the Radboud University Nijmegen approved the study.

### Operative procedure

Procedures were performed under semisterile conditions using a Zeiss operation microscope (Carl Zeiss AG, Oberkochen, Germany). Animals were anesthetized using a mixture of isoflurane, oxygen, and nitrogen, while breathing spontaneously through a mask. A midline laparotomy was performed, and in each rat, a 1-cm segment was resected from the descending colon 3 cm proximal to the peritoneal reflection. Colonic continuity was restored by constructing an end-to-end anastomosis with 8 single-layer, inverting, interrupted sutures (Ethilon 8-0; Ethicon, Norderstedt, Germany). A similar procedure was performed in the distal ileum, 15 cm proximal to the cecum. During operations, body temperature was kept at 38°C using a heating pad and a lamp. Intestines were covered with gauze pads soaked with 0.9% NaCl to minimize desiccation. To prevent dehydration, 10 ml of 0.9% NaCl was administered subcutaneously during the operation. If the animal was randomized into the fibrin glue group, 0.2 ml of fibrin glue (Tissucol Duo 500 0.5 ml; Baxter AG, Vienna, Austria) was applied around the anastomosis, immediately after its completion. All rats were administered with the analgesic buprenorphine (Temgesic; Schering-Plough, Houten, The Netherlands), 0.02 mg/kg, before operation and then every 12 h for 2 days after surgery.

### Wound strength

Sixty (30, control; and 30, fibrin glue) rats were killed using CO/CO_2_ asphyxiation on postoperative days 1, 3, or 5, and the abdomen was inspected. Segments containing the anastomoses were resected, and adhesions were dissected carefully without manipulation of the anastomosis, while fibrin glue was left in place. In order to measure the bursting pressure, the segments were infused (2 ml/min) with 0.9% NaCl containing methylene blue. The maximum pressure (mmHg) recorded immediately before the sudden loss of pressure was taken as the bursting pressure. The site of rupture (within or outside the anastomotic line) was noted. Subsequently, the segments were placed in a tensiometer, and the breaking strength (g) was measured [17]. The anastomotic segment was carefully cleaned from any adhering tissue and fibrin glue, and a 5-mm sample, containing the suture line in the middle, was frozen in liquid nitrogen and stored at −80°C until further processing.

### Biochemical analysis

After weighing, tissue samples were frozen, lyophilized, and pulverized. The hydroxyproline content, as a measure of the collagen content, was measured by high-performance liquid chromatography after hydrolysis with 6-N hydrochloric acid and coupling to dabsyl chloride.

Preparation of tissue extracts for gelatin zymography, using a buffer containing 1% (v/v) Triton X-100, has been described elsewhere [18]. The protein concentration of the extracts was measured using the bicinchoninic acid reagent. All tissue samples were stored at −80°C until zymography. The technique of preparation and electrophoresis of the gels and quantification of the various enzyme activities, which were expressed as arbitrary units on the basis of the lysed area, using a Sharp Jx-330 scanner and ImageMaster 1D software (Amersham Pharmacia, Uppsala, Sweden) have been described previously [18]. In-between comparison of values obtained on different gels was performed using collagenase type 1 (from *Clostridium histolyticum*; Sigma, St. Louis, MO, USA), as an internal standard. The presence of true matrix metalloproteinase (MMP) activity was confirmed by adding 10 mM EDTA or 1,10-phenanthroline to the buffers used after electrophoresis.

### Histology

The remaining 44 animals (see “Results”), divided evenly over the groups, were killed as described above at 1, 3, 5, or 7 days after surgery and used for histological evaluation. Adhesions were dissected carefully without manipulation of the anastomosis, while fibrin glue was left in situ. Intestinal samples of approximately 1 cm, containing the entire anastomosis in the middle, were carefully collected en bloc, opened at the mesenteric side, washed gently in 0.9%, and spread out in a cassette for paraffin embedding. From paraffin-embedded tissues, 4-μm sections were prepared and stained with hematoxylin and eosin (H&E). Sections were analyzed using a binocular light microscope.

Another set of longitudinal sections were stained with picrosirius red to identify collagen fibers in the anastomotic area, and collagen was quantified using digital image analysis [19]. Images were recorded using a 3-chip CCD RGB camera (DXC-325P, Sony) mounted on a conventional light microscope (Axioskop, Carl Zeiss), using a 5× objective. Image acquisition and analysis were performed using a complimentary software program (Zeiss KS 400® AxioVision 3.0). Microscopic images were digitized, and the area positive for picrosirius red staining was recognized by segmentation in RGB using fixed threshold values. The anastomotic area between the two inverted apposite muscular layers was interactively defined on the computer screen. The ratio of picrosirius red-positive and the total amount of pixels yielded the percentual area of anastomotic collagen.

### Statistics

Comparison between control and experimental groups was performed using a two-tailed unpaired *t* test or Fisher's exact test. Comparisons within one group (different time points) were performed using a one-way ANOVA followed by a Tukey–Kramer multiple comparisons test. Results were considered statistically significant at *p* < 0.05.

## Results

### General observations

**Fig. 1:**
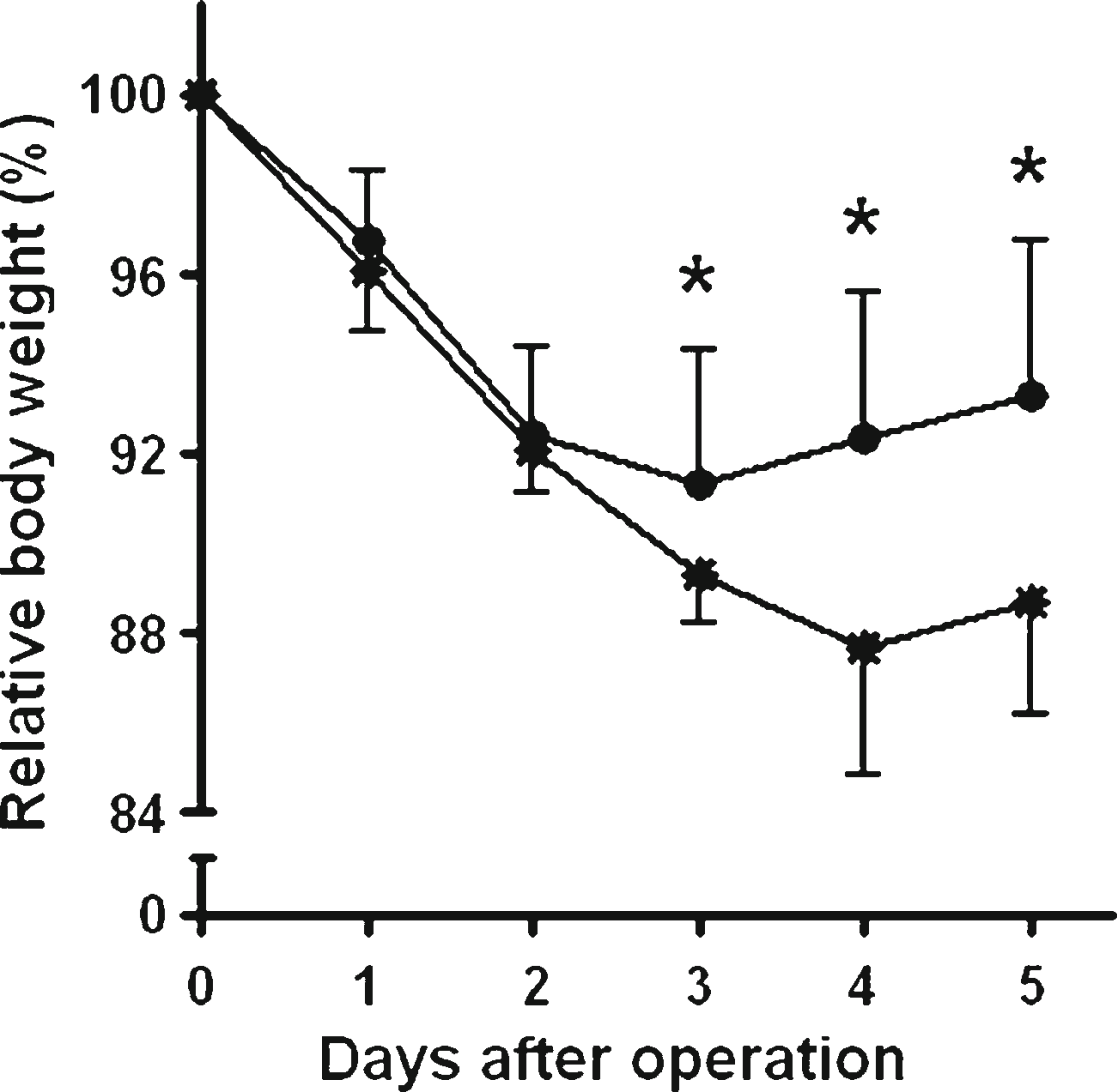
Postoperative course of body weight. Data represent mean (±SD) relative body weight, in relation to the weight prior to operation, for the control (*filled circle*, *n* = 16) and fibrin glue (*asterisk*, *n* = 15) groups. *p < 0.05 vs fibrin glue group

Three animals in the fibrin glue group did not survive surgery, while one animal in the control group died during the first night after operation. No abnormalities or signs of anastomotic leakage were seen in these animals. Up to day 3, the animals lost approximately 10% of their preoperative weight (Fig. 1). At day 3, the rats in the control group started to gain weight again, while the rats in the fibrin glue group decreased in weight up till 4 days after surgery. As a consequence, body weight in the latter group was significantly (*p* < 0.05) lower from day 3 onwards.

**Fig. 2:**
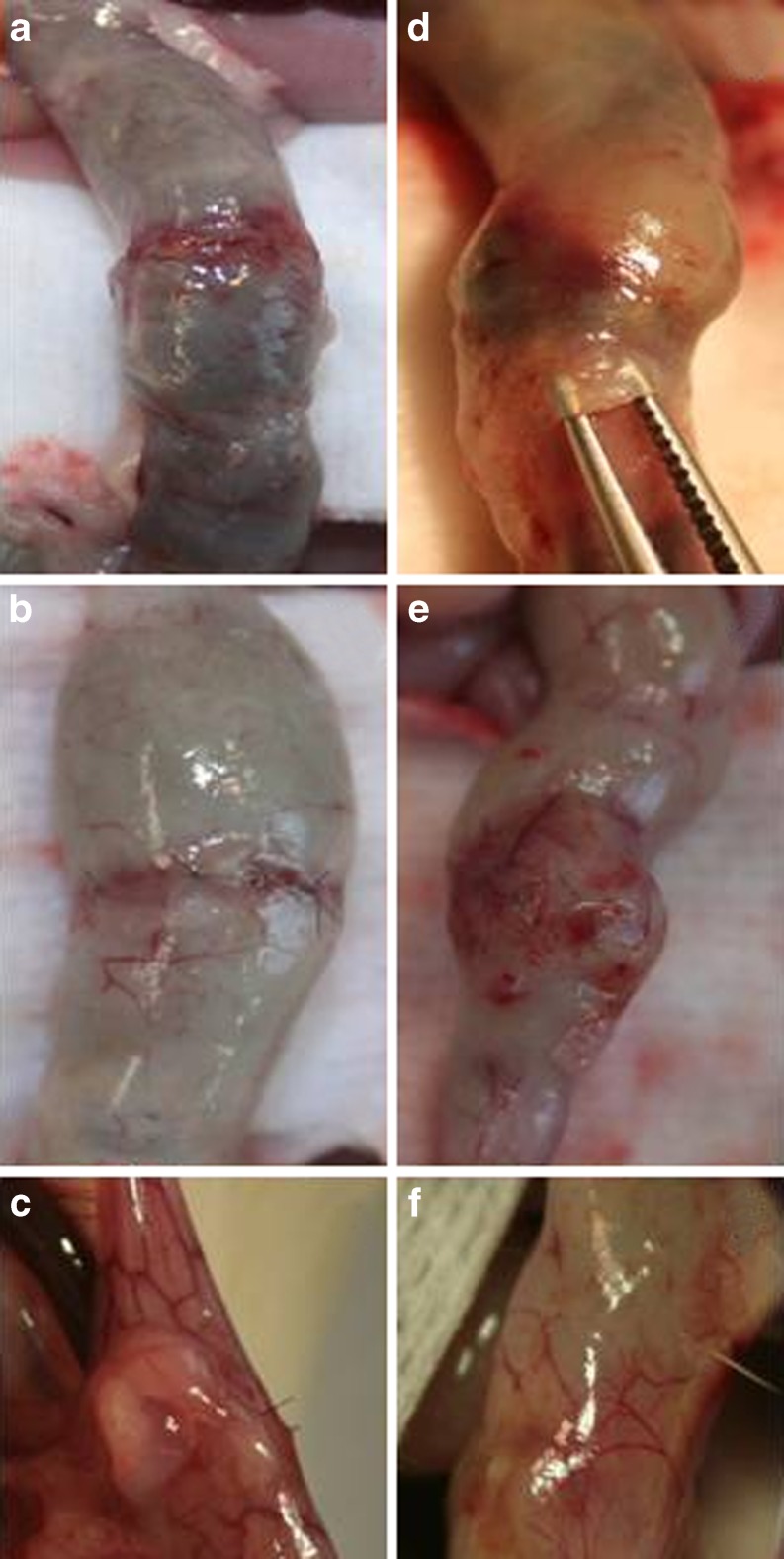
Macroscopic findings in the colonic anastomosis on days 1 (**a** and **d**), 3 (**b** and **e**), and 5 (**c** and **f**) in the control (*left side*) and fibrin glue (*right side*) groups

At day 3, 10 out of 16 rats from the fibrin glue group showed signs of a wide colon filled with hardened feces, immediately proximal to the anastomosis (Fig. 2e), which nevertheless remained conductant. This phenomenon, which was absent in the controls, was seen in only one rat at day 5 and in none of the rats on day 7. In addition, Fig. 2 represents the macroscopic findings at termination at days 1, 3, and 5 in the colonic anastomosis. On day 1, the fibrin glue was still present (lifted by the forceps on Fig. 2d), and on day 5, there were some adhesions to the fibrin glue (Fig. 2f), but adhesions were also present in the control group.

### Wound strength

**Fig. 3:**
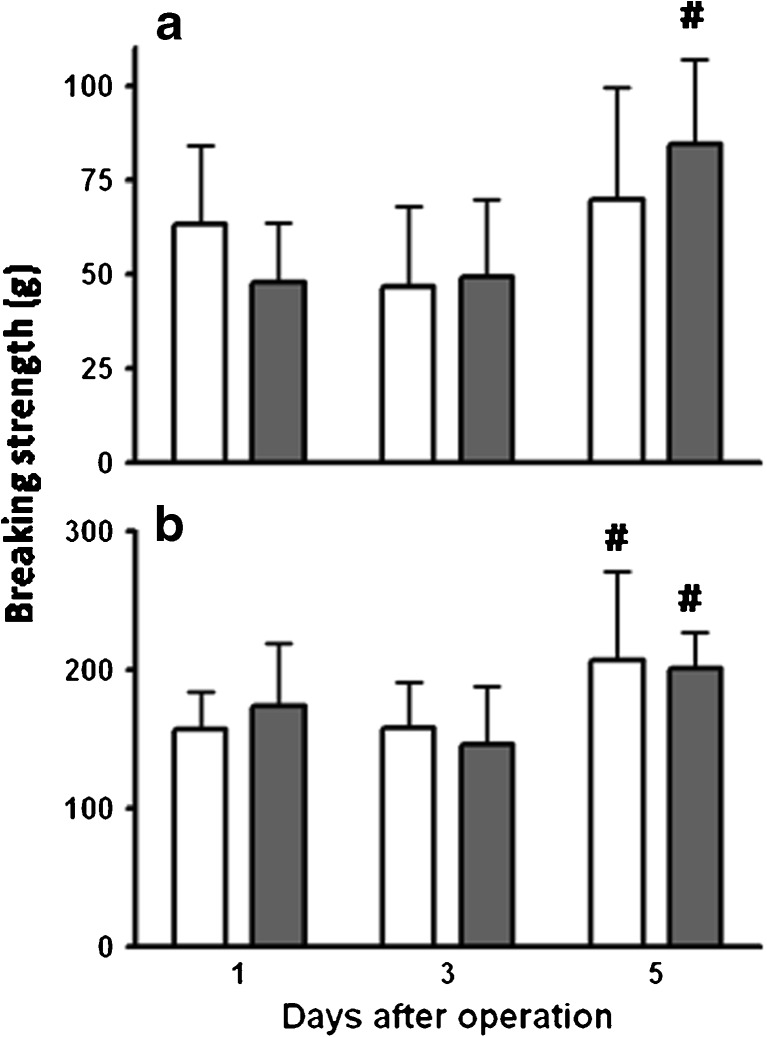
Anastomotic breaking strength. Data represent mean and SD for the strength in ileal (**a**) and colonic (**b**) anastomoses from the control group (*white bars*) and the group which received fibrin glue (*gray bars*). #*p* < 0.05 vs day 3

**Fig. 4:**
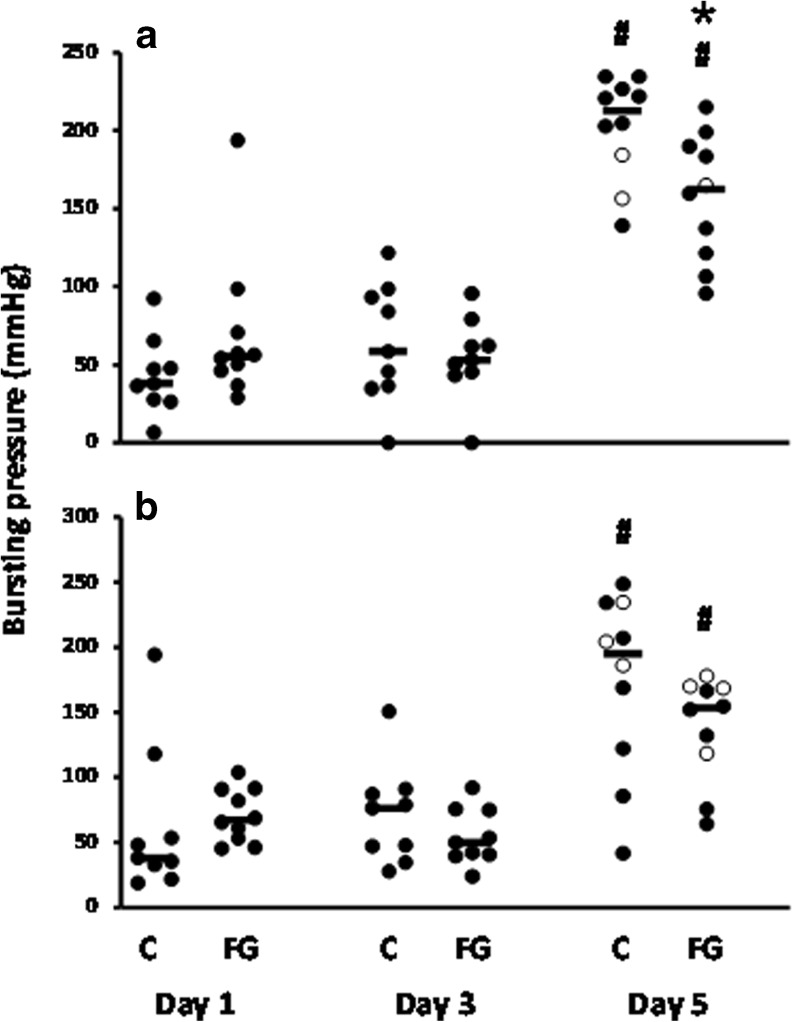
Anastomotic bursting pressure. Individual values and medians (*horizontal bars*) are given for ileal (**a**) and colonic (**b**) anastomoses in both control (*C*) and fibrin glue (*FG*) groups. The bursting site was either within (*filled circle*) or outside (*empty circle*) the suture line. *p < 0.05; #*p* < 0.05 vs day 3

The early anastomotic breaking strength remained low but increased in both groups between days 3 and 5 (Fig. 3). At all time points, average values in both groups were similar, both in the ileum and colon. Anastomotic bursting pressures remained low at day 1 and day 3, without any differences between the groups, and increased sharply thereafter (Fig. 4). From day 3 to day 5 in the ileum, the average bursting pressure was increased by a factor of 3.17 (*p* < 0.001) and 2.85 (*p* < 0.001) in the controls and fibrin glue-treated animals, respectively. In the colon, these factors were 2.44 (*p* < 0.001) and 2.51 (*p* < 0.001), respectively. However, at day 5, the bursting pressure in the fibrin glue group remained below than that in the controls, although only significantly (*p* = 0.0138) so in the ileum. Only at day 5, some bursting sites were outside the suture line (Fig. 4).

### Collagen and proteolytic activity

**Fig. 5:**
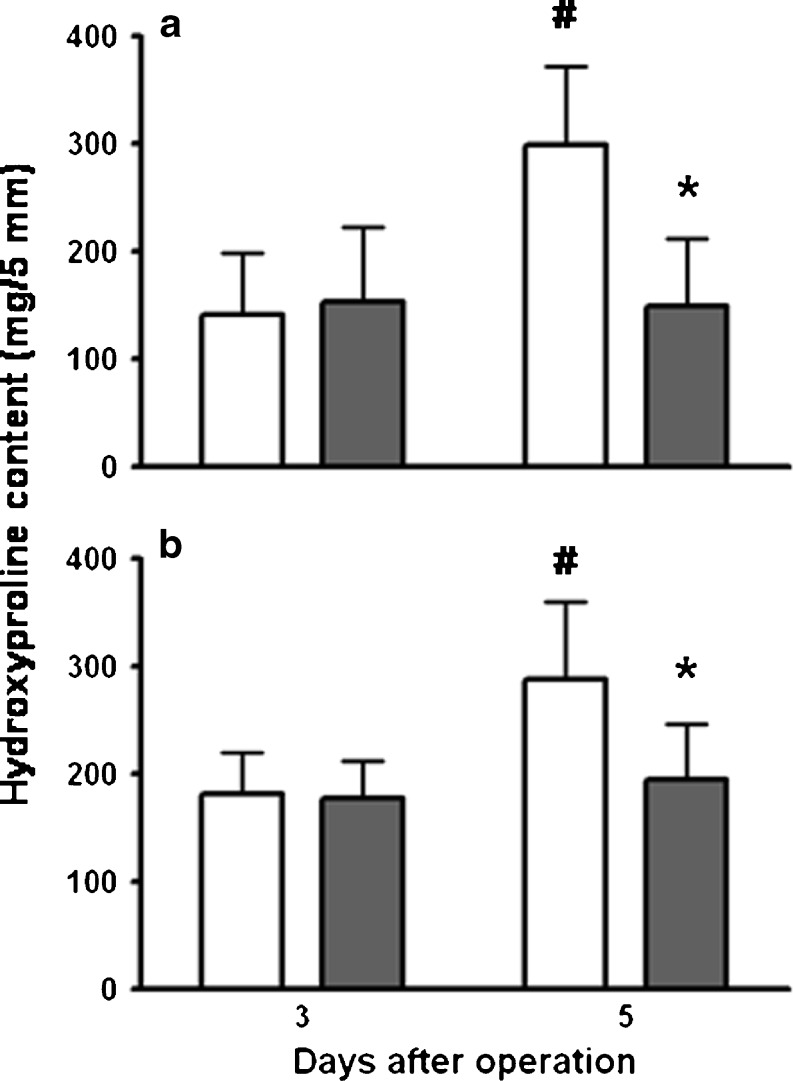
Hydroxyproline content of anastomotic segment. *Bars* represent mean and SD in ileal (**a**) and colonic (**b**) segments from the control group (*white bars*) and the group which received fibrin glue (*gray bars*). *p < 0.05 vs controls; #*p* < 0.05 vs day 3

**Fig. 6:**
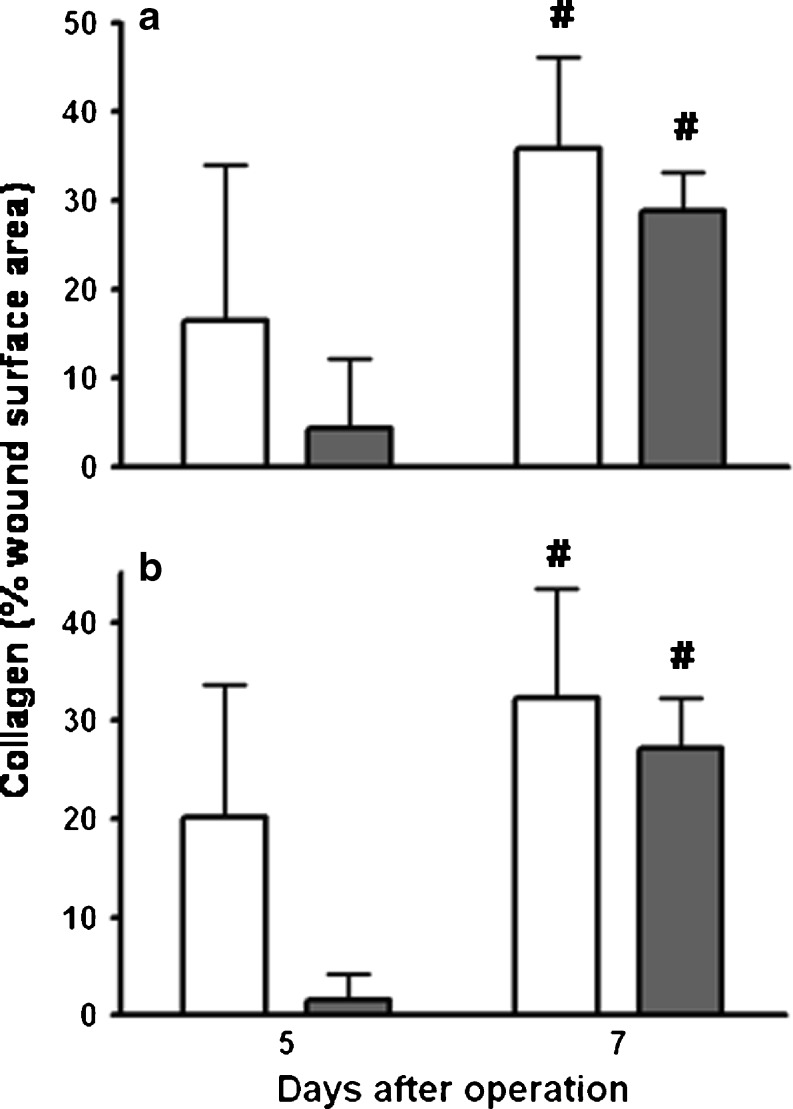
Wound collagen. *Bars* represent mean and SD in ileal (**a**) and colonic (**b**) segments from the control group (*white bars*) and the group which received fibrin glue (*gray bars*). #*p* < 0.05 vs day 5

In the controls, but not in the fibrin glue-treated animals, the hydroxyproline content in anastomotic segments increased significantly (*p* < 0.001) from day 3 to day 5 (Fig. 5). As a consequence, it was significantly lower in the fibrin glue group than that in the control group at day 5. This effect also appeared to be present if collagen was quantitated histologically in the true wound area, although not significantly (*p* = 0.054) so in the colon because of large inter-animal variations (Fig. 6). Still, at 7 days, no such effect was seen anymore as a result of the enormous increase observed in the fibrin glue group. Here, mean values for collagen as percentage of the wound surface area increased between days 5 and 7 from 4 to 29% (*p* = 0.0002) in the ileum and from 2 to 27% (*p* < 0.0001) in the colon.

Gelatin zymography of anastomotic extracts, obtained after 3 or 5 days, demonstrated the presence of multiple gelatin-degrading activities, representing proMMP-9, proMMP-2, and active MMP-2. No differences between the two experimental groups were found, with the exception of proMMP-9 in the ileum at day 3, where it was higher (*p* = 0.003) in the fibrin glue group than that in the controls (data not shown).

### Histology

**Fig. 7:**
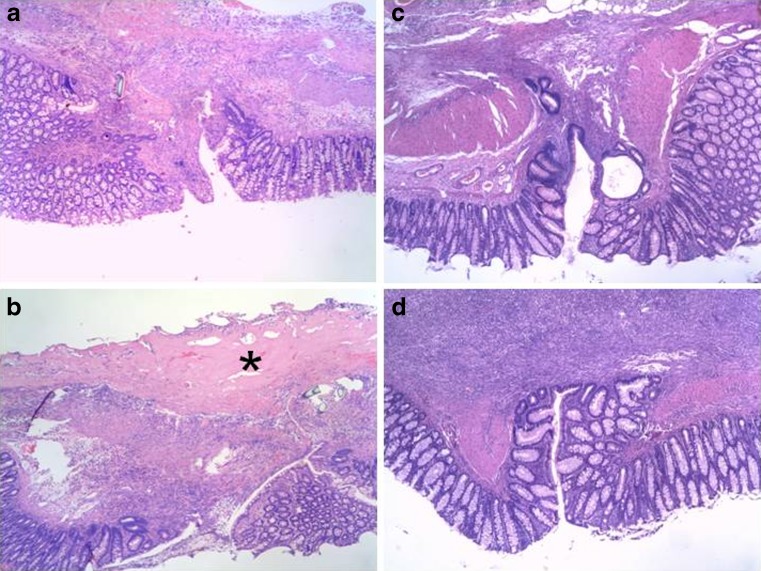
Anastomotic histology in the colon. Each panel shows a tissue segment with the anastomosis in the middle and the mucosal layer at the bottom at a magnification of approximately ×40, representing typical examples obtained at day 3 (**a**, control; and **b**, fibrin glue) and day 7 (**c**, control; and **d**, fibrin glue). The *asterisk* denotes fibrin

Typical examples of colonic anastomoses are presented in Fig. 7. No obvious or consistent differences in microscopic wound architecture were seen between groups. Fibrin glue was clearly present in the experimental group at day 3. At day 7, the fibrin glue appeared to be mostly gone. The same was observed in the ileum (not shown).

## Discussion

Anastomotic leaks are detected anywhere from 3 to 45 days postoperatively, and the diagnosis is mostly made between days 6 and 9 [20] or even later [21]. However, it stands to a reason that the processes which lead to the failure start much earlier, probably in the immediate postoperative period when the wound strength is believed to be low. The incidence and severity of the complication being as they are, it seems logical and even necessary to seek ways to protect the anastomotic integrity. There exists a renewed interest in staple or suture line reinforcement [3] with topically applied sealants, which is also reflected in recent preclinical studies [22–25].

Fibrin sealant is a well-established tool with multiple FDA-approved indications which are ever expanding [9]. Although its potential is often suggested, the application of fibrin glue to strengthen anastomoses in the gastrointestinal tract has not become a common practice. Mostly, its reported clinical application in gastrointestinal surgery seems to be limited to bariatric surgery [13] and treatment of fistulae [26, 27]. One question which needs an answer is if the application of fibrin glue, as a precaution against leakage, could be indicated for all intestinal anastomoses.

The present experimental data show fibrin glue to be not entirely harmless if applied to intestinal anastomoses. Application of fibrin around patent anastomoses in both the ileum and colon transiently affects repair. This effect presents clinically a temporary and resolving ileus and a delayed weight gain and, experimentally, a delay in collagen deposition in the true anastomotic area, which may cause the transient and relatively minor reduction in anastomotic bursting pressure.

Loss of body weight, always observed after operation, is more quickly compensated in the control group. In the fibrin glue group, signs of a wide colon filled with hardened feces, just proximal of the anastomosis, are seen on day 3, but not anymore at day 5. Possibly, fibrin glue can interfere with the peristaltic movement of the bowel, which leads to transient constipation in the large bowel only, because the consistency of the feces is different from the small bowel. Interestingly, ileus was also described after the application of collagen fleece which was kept in place by fibrin glue [25]. Early wound strength depends on the suture-holding capacity of the existing submucosal layer, while restoration of the original tissue strength depends on the deposition of newly made collagen fibrils. Interestingly, the application of fibrin glue appears to result in a reduced hydroxyproline content and a lessened presence of collagen fibrils in anastomotic segments at day 5. This phenomenon may explain the somewhat lower bursting pressures observed, although apparently sufficient collagen is still synthesized to cause the wound strength to increase strongly between days 3 and 5. Also, this is a transient phenomenon, since 1 week after operation, wound collagen deposition is similar in both groups. It is presently unknown how fibrin glue around the anastomosis would cause the slight delay in wound collagen formation, since fibrin is believed to be an excellent template for cellular migration [28].

The bursting pressure is a parameter for anastomotic strength, only if, the bursting site is within the suture line, which will mostly not be the case anymore at day 7 after operation. For that reason and the fact that no differences in the breaking strength are seen between the groups up until day 5, we only assessed the histology after 7 days.

The present data extend earlier, limited, findings where a significant loss of colonic bursting pressure was described at days 4 [16] and 5 [15] after surgery. In our study, the bursting pressure at day 5 is still quite high and much elevated, compared to day 3. Still, a certain delay in the recovery of intestinal strength cannot be denied, while wound strength during the first 3 days appears unaffected. Altogether, the available evidence indicates fibrin glue to be not beneficial, and at its best irrelevant, to anastomotic healing in the intestine.

The conclusion must therefore be that there is no justification for using fibrin glue on patent anastomoses constructed under low-risk conditions. It remains to be determined if it can play a beneficial role in other situations. In a very recent study on partial colonic anastomoses constructed with a limited number of sutures, a positive effect of fibrin glue was observed on the anastomotic bursting pressure at days 3 and 5 after operation [29]. Clinically, its application over stapled anastomoses during laparoscopic resection of rectal cancer has been reported, where a declining tendency of the anastomotic leakage was suggested [14]. Thus, it may be that it can play a protective role under conditions where chances for anastomotic leakage are enhanced. Under those conditions, its potential benefit may outweigh its unwanted effects.
